# Latency Associated Peptide Has *In Vitro* and *In Vivo* Immune Effects Independent of TGF-β1

**DOI:** 10.1371/journal.pone.0001914

**Published:** 2008-04-02

**Authors:** Naeem A. Ali, Alice A. Gaughan, Charles G. Orosz, Chris P. Baran, Sara McMaken, Yijie Wang, Timothy D. Eubank, Melissa Hunter, Frank J. Lichtenberger, Nicholas A. Flavahan, Jack Lawler, Clay B. Marsh

**Affiliations:** 1 Department of Internal Medicine, The Ohio State University, Columbus, Ohio, United States of America; 2 Department of Surgery, The Ohio State University, Columbus, Ohio, United States of America; 3 The Dorothy M. Davis Heart and Lung Research Institute, The Ohio State University, Columbus, Ohio, United States of America; 4 Department of Anesthesiology and Critical Care Medicine, The Johns Hopkins University, Baltimore, Maryland, United States of America; 5 The Department of Pathology, Beth Israel Deaconess Medical School and Harvard Medical School, Boston Massachusetts, United States of America; New York University School of Medicine, United States of America

## Abstract

Latency Associated Peptide (LAP) binds TGF-β1, forming a latent complex. Currently, LAP is presumed to function only as a sequestering agent for active TGF-β1. Previous work shows that LAP can induce epithelial cell migration, but effects on leukocytes have not been reported. Because of the multiplicity of immunologic processes in which TGF-β1 plays a role, we hypothesized that LAP could function independently to modulate immune responses. In separate experiments we found that LAP promoted chemotaxis of human monocytes and blocked inflammation *in vivo* in a murine model of the delayed-type hypersensitivity response (DTHR). These effects did not involve TGF-β1 activity. Further studies revealed that disruption of specific LAP-thrombospondin-1 (TSP-1) interactions prevented LAP-induced responses. The effect of LAP on DTH inhibition depended on IL-10. These data support a novel role for LAP in regulating monocyte trafficking and immune modulation.

## Introduction

Transforming Growth Factor-Beta 1 (TGF-β1, NM_011577) has diverse effects in multiple cell types. It is intimately involved in cell growth, differentiation, and immune modulation. [1], [2] Pathologic activation of TGF-β1 is associated with the development of fibrosis[3]–[5] while deficiency in TGF-β1 is associated with increased inflammatory cell trafficking and inflammation.[6], [7] As a result, TGF-β1 is characterized as an anti-inflammatory and pro-fibrotic growth factor. Curiously, while TGF-β1 is considered an anti-inflammatory cytokine, it induces leukocyte recruitment.[8], [9]

TGF-β1 activity is controlled predominantly through activation of the latent molecule. After post-translational processing, TGF-β1 binds non-covalently to the latency associated peptide (LAP) to confer latency.[10] This small latent complex exists without or with latent TGF-β1 binding protein (LTBP), which is involved in the release and targeting of TGF-β1 to the extracellular matrix.[10], [11] The non-covalent interactions between these molecules can be disrupted by heat, extremes of pH and other chaotropic factors *in vitro*, but *in vivo*, disruption of the physical interactions between LAP and TGF-β1 appears central to activation.[12], [13] Because of its critical role in modulating TGF-β1 activity, LAP plays a pivotal role in regulating some of the diverse effects of TGF-β1. In fact, LAP has been shown to be expressed on immature dendritic cells and play a role in T cell differentiation.[14] Previous work shows that LAP can induce epithelial cell migration,[15], [16] but effects on leukocyte recruitment have not been reported. Using an animal model of skin fibrosis with associated inflammation, *in vivo* treatment with LAP abrogates fibrosis but does not affect leukocyte infiltration[5], raising the possibility that LAP may independently stimulate inflammatory cell recruitment.

We hypothesized that LAP independently modulated immune cell function. Using *in vivo* murine models and *in vitro* human cell studies, we found that LAP had both chemotactic and anti-inflammatory activity independent of active TGF-β1.

## Methods

### Cell isolation

#### Monocyte isolation

Freshly isolated human peripheral blood monocytes were used for these experiments. Human blood samples were collected from healthy volunteer donors. Freshly drawn whole blood or buffy coat preparations were used and the isolation was performed as described previously.[17] Monocytes used in chemotaxis assays were maintained in media (RPMI with 5% FBS) until the experiment was performed and then they were washed and suspended in Gey's Balanced Salt Solution (GBSS) (Sigma-Aldrich, St Louis, MO) without serum. All monocyte suspensions were treated with polymyxin B at 10 µg/ml. (Sigma-Aldrich, St Louis, MO)

#### Macrophage Isolation and Culture

Bone marrow-derived macrophages (BMM) were obtained from *thrombospondin-1 −/−* (MGI:98737)or wild-type C57BL/6 mice. Briefly, bone marrow progenitor cells were flushed out of the bone marrow using ice-cold RPMI medium. The resulting isolate was then plated in RPMI supplemented with 10% FBS, penicillin/streptomycin and amphotericin B, 10 µg/ml polymyxin B and 20 ng/ml M-CSF. Cells were cultured in 37°C for 5 days with the addition of 20 ng/ml M-CSF daily. After twenty-four hours, non-adherent cells were removed and the remaining cells were cultured to generate macrophages. After isolation, BMM were serum starved for 12 to 16 hours at 37° C before being used for chemotaxis studies.

### Cellular recruitment assays

#### Matrigel plug assay

Six-week-old C57BL/6 female mice were anesthetized with isoflurane and subcutaneously injected with 0.5 ml growth factor-reduced Matrigel™ (BD Biosciences/Discovery Labware, San Jose, CA) matrix supplemented with either PBS (negative control for chemotaxis), 10 ng/ml CCL2 (positive control for chemotaxis) or 10 pg/ml of rhLAP. After 10 days the mice were sacrificed, skinned, and the Matrigel™ plugs were removed and placed in formalin for 24 hours. After, the formalin was discarded and replaced with PBS. The samples were paraffin-embedded and three sections per plug were cut and adhered to slides for subsequent staining using Hematoxylin & Eosin (H&E) for total cell influx into the plugs, stained for CD68+ cells (mononuclear phagocytes) with the respective isotype control antibody stains done as well. Ten high power fields were photographed per condition and CD68+ cells counted in a blinded fashion.

#### Recruitment chamber assay

A 48-well chemotaxis chamber (Neuroprobe, Rockville, MD) was used for all chemotaxis assays. Recombinant human latency associated peptide (LAP, R&D Systems, Minneapolis, MN) was cleared of endotoxin by the use of END-X B15 beads (endotoxin-binding resin, Woods Hole Associates, Woods Hole, MA) and suspended in media containing polymyxin B (10 µg/ml) (Sigma-Aldrich, St Louis, MO). TGF-β1 used was also obtained from R&D Systems and was tested in systems with polymyxin B. The tested solution (28 µl) was loaded into the bottom well and a monocyte suspension (1×10^6^ cells/ml) was added to the upper chamber (45 µl). The chamber was incubated at 37° C and 5% CO_2_ for 90 minutes. Experimental conditions were performed in at least triplicate.

Chemotaxis was measured on a 5-micron pore polycarbonate filter specifically designed for chemotaxis assays using modified Boyden chambers (GE Osmonics, Minnetonka, MN). The filters were then removed, fixed and stained in Diff-quik® solution (Fisher Scientific, Fairlawn, NJ) and viewed under a microscope. Five blinded high-power fields were counted, averaged, and reported as the number of monocytes/high power field. These methods are as previously published for TGF-β1-induced monocyte chemotaxis.[9]

In some studies, recombinant human LAP (rhLAP) or active TGF-β1 (R&D Systems, Minneapolis, MN) was incubated with TGF-β1, rhLAP, anti-LAP, anti-TGF-β1 antibodies or isogenic control (all antibodies 1 µg/ml) for thirty minutes prior to the addition of the monocyte suspension. For other inhibitor studies looking at interactions with LAP that were tested in chemotaxis assays, the monocyte suspension was incubated with the appropriate antibody (1 µg/ml), peptide (20 µM LSKL and GRGDSP), signal inhibitor (U0126, 5 µM and SB 431542, 5 µM and 10 µM) or control (isotype antibody at 1 µg/ml or scrambled peptide, 20 µM SLLK and GRGESP) for thirty minutes prior to washing and using in chemotaxis studies.

#### Directed-recruitment (Chemotaxis) assay

Similar experiments were performed to the recruitment assays described above, except that the potential gradient of LAP was obliterated with adding equal amounts of LAP to both the upper well (monocyte suspension) and lower well (chemoattractant chamber). This was performed to assure that monocyte migration mediated by LAP was chemotaxis. In these studies a concentration of the tested agent (LAP) was added to the lower well only or to the upper and lower well. The monocyte suspension was placed in the upper well only. If a non-specific increase in cell motility was the true response of monocytes to exposure to LAP, increased monocyte numbers would still be seen in the LAP exposed monocytes despite the existence of a zero effective gradient to direct migration.

#### Inhibitors

LSKL and SLLK were kindly provided by Pravin Kaumaya (The Ohio State University) while RGD blocking peptide (G4391, GRGDS) and scramble control peptide (A5686, RGES acetate salt) were from Sigma-Aldrich (St Louis, MO) and LAP-derived RGD sequence (GRGDSP) and control peptide (GRGESP) were from Invitrogen (Carlsbad, CA). Anti-LAP (MAB-246, clone 27235.1) and anti-TGF-β1 (MAB-240) antibodies were from R&D systems (Minneapolis, MN). Anti-CD36 IgG (Ab-2, clone 185-162) and anti-CD47 (Ab-2, clone B6H12.2) were from Neomarkers (Fremont, CA). Thrombospondin-1 antibody (clone A6.1, BA-24) was from Calbiochem (Fremont, CA). Polyclonal mouse IgG (polyclonal mouse IgG, sc-2025) was from Santa Cruz Biotechnology, Santa Cruz, CA. SB 431552 was from Sigma-Aldrich (St Louis, MO). Finally, U0126 was from Calbiochem (Fremont, CA).

### Murine delayed-type hypersensitivity response transfer assay

We previously published a method of measuring *in vivo* delayed type hypersensitivity using a mouse model of antigen and immune response transfer that is inhibited by TGF-β1.[18] Wild-type mice (C57BL/6) were tested for DTH responses using a transfer DTH assay. For this assay, the pinnae of naïve C57BL/6 mice were injected using a 30-gauge insulin syringe with 35 µl of a mixture containing 8×10^6^ splenocytes from tetanus toxin-sensitized mice plus tetanus toxin with or without 5 ng of porcine TGF-β1 (R&D Systems) or 10 pg of human LAP (R&D Systems). Alternatively, this assay was repeated using C57BL/6 mice that had rejected a cardiac allograft (DBA/2->C57BL/6, “rejector” mice) as a model of allograft DTHR. This model has been shown to yield similar results to the DTH model.[19] DTHR was induced in the pinnae of naïve C57BL/6 mice with simultaneous injection of subcellular DBA/2 alloantigen (35 µl) and splenocytes (8×10^6^cells/condition) harvested from these sensitized “rejector” mice between 30–60 days post transplant. In other experiments, 10 µg of anti-thrombospondin-1 rabbit polyclonal antibodies (Neomarkers, Fremont, CA), 25 µg of anti-IL10 goat polyclonal antibodies (BD Biosciences) or isogenic control IgG were also included in the DTH injection mixture.

Alternatively, tetanus-sensitized C57BL/6 *thrombospondin-1 -/-* mice were tested for DTH responses between 14–28 days post-tetanus sensitization using a direct DTH assay. For this assay, the pinnae of the sensitized mice were injected using a 30-gauge insulin syringe, with a 35 µl mixture of 25 µl (limit of flocculation) of tetanus toxoid (Adventis, Stillwater, PA) ±5 ng of porcine TGF-β1 (R&D Systems) or 10 pg of rhLAP (R&D Systems). Changes in ear thickness for both sets of experiments were measured both before injection and 24 hours after injection using a dial thickness gauge (Swiss Precision Instruments, Carlstadt, NJ). For reference, changes in the range of 0–30 ×10^−4^ inches represent background swelling due to injection trauma, changes in the range of 40–60 ×10^−4^ inches represent moderate DTH response, and changes in the range of 70–100 ×10^−4^ inches represent strong DTH responses.[20]

### Macrophage stimulation

Murine macrophages (RAW 264.7 cells) were grown in sterile conditions. Wells were then washed to remove all non-adherent cells and serum starved overnight at 37°C. The cells were then stimulated with equal molar of LAP (100ng/ml), TGF-β1 (36ng/ml), latent TGF-β1 (121ng/ml) or control M-CSF (100 ng/ml) for 10 minutes. Cells were lysed, protein quantitated, and Western Blotted for phospho-Smad 2/3 (Cell Signaling Technology, Danvers, MA), then re-probed for total Smad 2/3.

### Statistical analysis

For all studies, one-way ANOVA was used to test for significant (p-value of <0.05) differences in the analyzed data. Post-hoc analysis was used to differentiate individual differences reflected in the group. Tukey's pairwise comparison was used for chemotaxis assays, while Fisher's pairwise was used for DTHR transfer assays. All analyses were run on Minitab® statistical software (State College, PA).

All human studies were approved by the Ohio State University Biomedical IRB. Animal studies were performed only after approval of the research plan by the Institutional Animal Care and Use Committee at The Ohio State University.

## Results

### LAP is a monocyte chemoattractant

**Figure 1:**
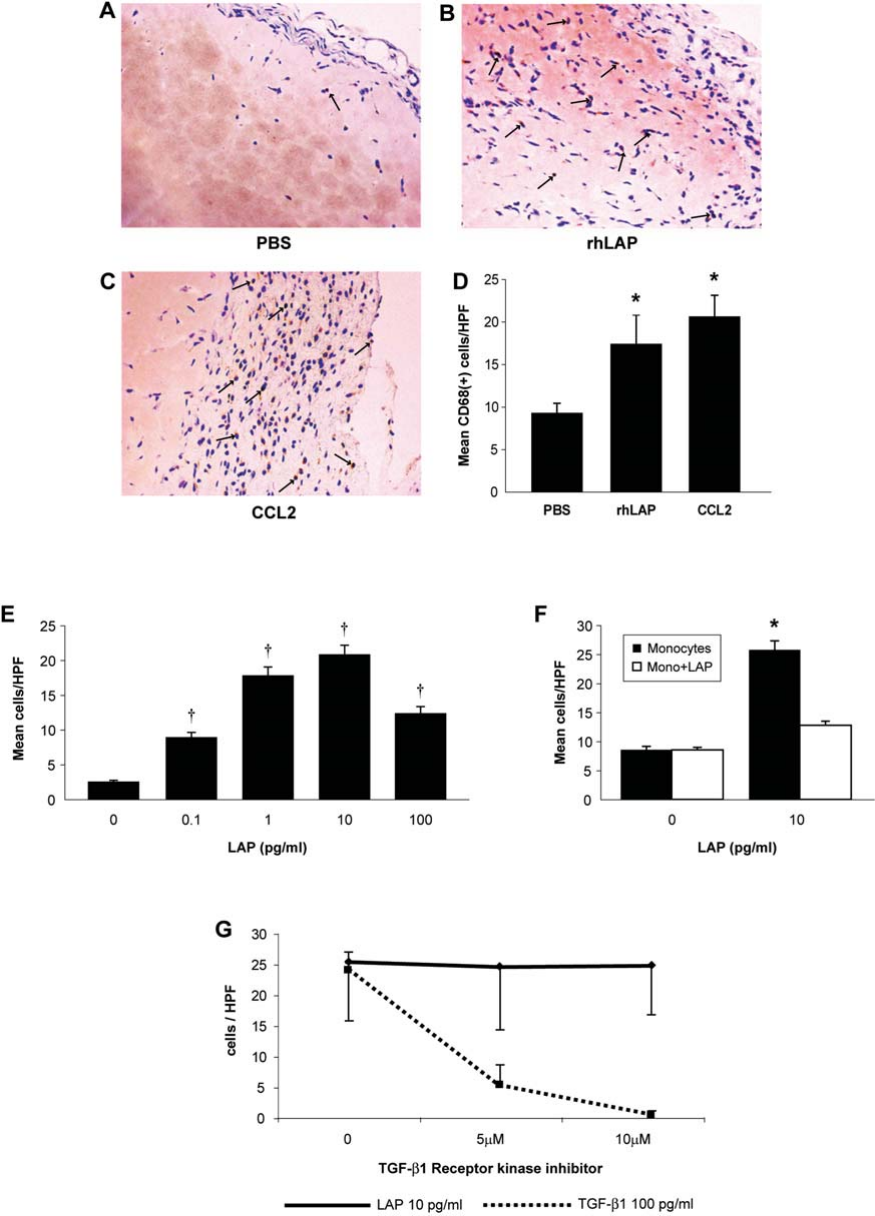
Latency-associated peptide (LAP) induces monocyte chemotaxis via a pathway distinct from TGF-β1. C57BL/6 female mice were anesthetized and subcutaneously injected with Matrigel™ matrix supplemented with PBS, rhLAP or CCL2/MCP-1. After 10 days the mice were sacrificed, skinned, and the plugs were removed, fixed and embedded in paraffin then stained for CD68+ cells (mononuclear phagocytes). Matrigel matrix supplemented with (A) PBS, (B) 10 pg/ml rhLAP, or (C) 10 ng/ml CCL2 were assessed for CD68+ cell recruitment and then quantified by blinded counts (D). Arrow heads indicate CD68+ cells. (*p<0.05 for condition vs. PBS control plugs, n = 3 for CCL2 and n = 2 for LAP) Human monocytes (5×10^4^/condition) were suspended in Gey's balanced salt solution and assayed for their chemotactic response to rhLAP. Recruitment was assayed by incubating monocytes in modified Boyden chambers for 90 minutes at 37°C and quantified by counting five high-powered fields of stained membranes in a blinded fashion. (E) Increasing doses of rhLAP (0-100 pg/ml) were used to stimulate monocyte recruitment (†p<0.001 from the non-stimulated sample, n = 7). (F) Analysis for evidence of directed recruitment of monocytes by LAP was assessed by exposing monocytes to a “true” gradient of rhLAP (10 pg/ml) (rhLAP in the chamber opposite the monocytes *only*; dark bars–“Monocytes”) or no gradient (rhLAP on both sides of the chamber; lighter bars–“Monocytes+LAP”). These data represent two independent studies done in triplicate (*p<0.001 vs. mono + LAP condition). (G) Assessment of LAP-specific chemotaxis was performed by pre-incubating cell suspensions with the SB 431542 hydrate from Sigma (St.Louis, MO), a selective inhibitor of TGF-β type 1 receptor kinases (Activin Receptor-Like Kinases, ALK-4,-5, and-7) 20 minutes prior to loading on chemotaxis chambers. Monocytes were then exposed to optimal chemotactic doses of LAP (10 pg/ml) and TGF-β1 (100 pg/ml). All bars represent the mean±SEM for n = 4 samples (* p<0.05 by ANOVA with post-hoc testing).

To evaluate the biological effects of LAP, we first studied monocyte chemotactic activity. To test this *in vivo,* Matrigel™ plugs impregnated with LAP were inserted into the subcutaneous tissues of mice. LAP-supplemented plugs recruited more CD68 positive cells than control plugs injected with PBS (Figure 1A–D). To understand this observation, *in vitro* studies of human monocyte chemotaxis to LAP were performed. Recombinant human LAP induced a dose-dependent increase in monocyte recruitment compared to vehicle-treated monocytes (p<0.001) (Figure 1E). Monocyte recruitment occurred when a true gradient of LAP (p<0.001) existed, but was abrogated by equilibrating LAP concentrations across the chemotaxis membrane, suggesting that LAP promoted monocyte chemotaxis and not chemokinesis (Figure 1F). In addition, human monocytes did not migrate to an irrelevant stimulus like the neutrophil chemoattractant, IL-8 (3.2±0.5 cells/high powered field for IL-8 vs. 2.7±0.37 cells/high powered field for media alone [mean±SEM]) and vigorously responded to a positive control like CCL2 under both anti-LAP antibody-treated (64.4±3.7 cells/high powered field [mean±SEM]) and control IgG-treated conditions (63.6±3.6 cells/high powered field [mean±SEM]). Interestingly, at 0.4 pM (10 pg/ml equivalent) equimolar concentrations of CCL2 and LAP, LAP induced about 2-fold more monocyte chemotaxis than CCL2, but at 1.2 µM (10 ng/ml) equimolar concentrations, CCL2 was significantly better at inducing monocyte chemotaxis than LAP (data not shown).

### LAP-induced chemotaxis is not mediated by TGF-β1

Even though LAP used in these studies was a recombinant human protein, to ensure that monocyte chemotaxis induced by LAP did not involve endogenous active TGF-β1, we assessed whether a variety of TGF-β1 inhibitors could affect LAP-induced chemotaxis. Chemotaxis to LAP was blocked by anti-LAP antibodies (Table 1, p<0.05), but not by equivalent concentrations of anti-TGF-β1 neutralizing antibodies. In contrast, monocyte chemotaxis induced by active TGF-β1 was inhibited by anti-TGF-β1 antibodies, but not by anti-LAP antibodies. Isogenic control antibodies did not suppress LAP- or TGF-β1-mediated chemotaxis. Finally, using a specific receptor kinase inhibitor, we show that inhibiting TGF-βR1 signaling abrogates TGF-β1-induced monocyte recruitment without affecting LAP-induced chemotaxis. (Figure 1G) Separately, using bone-marrow derived macrophages we were able to show that LAP did not induce SMAD activation, whereas TGFβ1 did (data not shown). These data confirm that LAP has chemotactic activity for human monocytes *in vitro* independent of TGF-β1.

**Table 1:** LAP-induced monocyte chemotaxis is blocked by specific inhibitors of LAP-TSP-1 interactions.

Inhibitor Treatment of Monocytes	Fold-change in monocyte recruitment to LAP vs. “Untreated” control	*P value*
Untreated	4.3±1.0	
Anti-LAP IgG	1.3±0.4	***<0.05***
Anti-TGF-β1 IgG	4.3±0.7	***ns***
anti-TSP-1 IgG	1.1±0.1	***<0.01***
anti-CD36 IgG	1.2±0.1	***<0.001***
anti-CD47 IgG	6.2±0.7	***ns***
Isogenic Control Antibody	3.0±0.5	***ns***
LSKL	0.9±0.2	***<0.01***
SLLK	3.3±0.5	***ns***
RGD-blocking peptide	6.5±0.6	***ns***
Scramble RGD-blocking peptide	8.2±2.4	***ns***
UO126 MEK inhibitor	1.2±0.3	***<0.01***

Human monocytes (5×10^4^/condition) were suspended in Gey's balanced salt solution and assayed for their chemotactic response to rhLAP. Recruitment was assayed by incubating monocytes in modified Boyden chambers for 90 minutes at 37°C and quantified by counting five high-powered fields of stained membranes in a blinded fashion and reported as fold-increase of LAP (10 pg/ml)-induced monocyte chemotaxis compared to monocyte chemotaxis induced by media alone. rhLAP at 10 pg/ml was chosen based on previous experiments that determined this dose induced maximal cellular recruitment. These data represent n = 3 (minimum) separate experiments expressed as the mean fold-change±SEM. Reported p values compare fold-change in monocyte chemotaxis induced by LAP without and with identified inhibitors.

### TGF-β1 can inhibit LAP-induced monocyte chemotaxis

**Figure 2:**
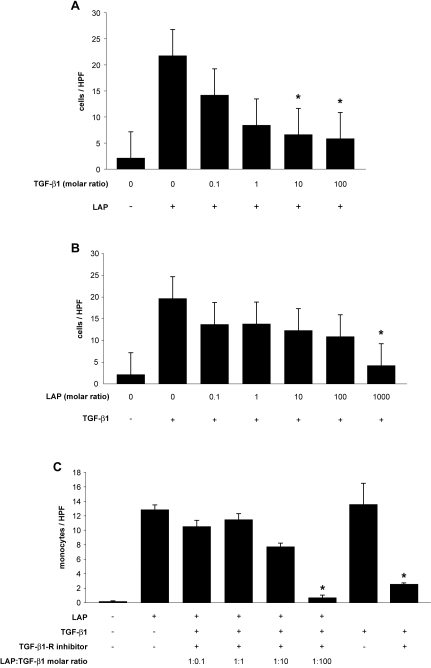
TGF-β1 is able to inhibit LAP-induced monocyte recruitment. (A) 10 pg/ml of rhLAP (dose with maximal response) was used as the chemoattractant for freshly isolated human peripheral blood monocytes in chemotaxis chambers and assessed as in previous experiments. Increasing doses of active rhTGF-β1 were pre-incubated with the chemoattractant prior to exposure to the monocyte suspensions (*, p<0.05 vs. LAP alone). (B) To compare this to the activity of LAP as an inhibitor of active rhTGF-β1 function in this system, we performed parallel experiments where the conditions were reversed. Active rhTGF-β1 was used at its maximal effective dose (100 pg/ml) based on prior experiments (data not shown). (*, p<0.05 vs. TGF-β1 alone) All bars represent the mean±SEM. (C) Assessment of LAP-mediated recruitment independent of TGF-β1 receptor activity in these experiments was assayed using SB 431542 hydrate, a selective inhibitor of TGF-β type 1 receptor kinases (TGF-β1-receptor inhibitor). Cell suspensions were preincubated for 20 minutes with 10 µM SB 431542 (optimal dose blocking TGF-β−induced signaling) or DMSO. 10 pg/ml of rhLAP (dose with maximal response) was used as the chemoattractant for freshly isolated human peripheral blood monocytes in chemotaxis chambers and assessed as in previous experiments. Increasing doses of active rhTGF-β1 were pre-incubated with the chemoattractant prior to exposure to the monocyte suspensions (*, p<0.05 vs. LAP alone) (*, p<0.05 vs. TGF-β1 alone). All bars represent the mean±SEM for n = 3 samples.

In preliminary experiments we discovered that a 1∶1 molar ratio of TGF-β1 and LAP reduced monocyte recruitment (data not shown). Knowing that LAP *in vitro* limits TGF-β1 activity, we hypothesized that TGF-β1 functioned similarly toward LAP activity. To explore this further, we performed chemotaxis assays using either TGF-β1 or LAP at their maximal recruitment doses alone or with increasing molar ratios of its partner. We identified that active TGF-β1 inhibited LAP-induced monocyte chemotaxis (Figure 2A) just as LAP could also inhibit TGF-β1-induced monocyte chemotaxis (Figure 2B). Interestingly at peak chemotactic doses, TGF-β1 was a more potent inhibitor of LAP (10∶1 molar ratio) than LAP was of TGF-β1 (1000∶1 molar ratio). To confirm that TGF-β1 inhibition of LAP-induced recruitment was independent of TGF-β receptor activity we repeated these experiments in the presence of a TGF-β receptor inhibitor (Figure 2C). The experiments demonstrate that TGF-β1 induced inhibition of LAP is not mediated through the TGF-β1 receptor.

### The tetrapeptide LSKL, anti-thrombospondin-1 antibodies, and the MEK inhibitor block LAP-induced monocyte chemotaxis

Integrins often play a role in cellular migration.[21] To investigate the role of integrins in monocyte migration to LAP, experiments using RGD blocking peptides to modulate cellular recruitment were performed. RGD blocking peptides did not reduce LAP-mediated chemotaxis (Table 1). Because thrombospondin-1 interacts with LAP to regulate TGF-β1 function,[13], [22] we performed experiments to determine if these interactions were important in regulating the biologic activity of LAP. To determine the importance of TSP-1 in LAP-induced monocyte recruitment, anti-TSP-1 antibodies were used. Anti-TSP-1, but not isogenic control IgG, blocked monocyte recruitment to LAP (Table 1) (p<0.01). Because CD36 is expressed on human monocytes and interacts with TSP-1, anti-CD36 antibodies were tested to determine its role in LAP-induced cellular recruitment. Anti-CD36 IgG, but not isogenic control IgG, blocked monocyte recruitment to LAP (Table 1) (p<0.001). CD47 associates with integrins and binds the c-terminal region of TSP-1 so its role was explored. Similar to RGD peptides, pre-incubation with CD47-blocking IgG did not affect LAP-mediated monocyte chemotaxis (Table 1). Control experiments demonstrated that none of the inhibiting antibodies affected the monocyte response to CCL2, providing support that the inhibitory pathway tested was specific to an LAP-mediated process (data not shown). These data confirmed that TSP-1 and CD36 played a role in the chemotactic activity of LAP.

To further define the role of TSP-1 in LAP stimulation, known sites of direct interaction between LAP and TSP-1 were explored. Two important sites of interaction on TSP-1 that interact with latent TGF-β1 are contained in the thrombospondin type 1 repeat (TSR) region of TSP-1.[23], [24] One of these regions, the tetrapeptide motif K^412^RFK^415^ is important for latent TGF-β1 activation and interacts directly with a defined region of LAP (L^54^SKL^57^).[25] To analyze this specific interaction between LAP and TSP-1 in monocyte chemotaxis, the synthetic peptide LSKL was used as a domain specific competitive inhibitor of these two molecules. When monocytes were pre-incubated with LSKL, monocyte chemotaxis to LAP was reduced (p<0.01), while equivalent concentrations of the scrambled peptide SLLK did not affect LAP-induced monocyte chemotaxis (Table 1). These data further support the importance of the interaction between LAP and TSP-1 in LAP-induced chemotaxis.

Because CD36 inhibition effectively blocked LAP-induced chemotaxis and TSP-1 engagement produces activation of the MAPK pathway[26], we used a MEK inhibitor (U0126) in a functional chemotaxis assay with LAP. We found that U0126 inhibited LAP-induced chemotaxis (p<0.01) where DMSO alone did not **(** Table 1 **)** suggesting that the Erk/MAPK pathway was relevant to LAP-induced monocyte activation.

### LAP reduces swelling in a DTHR murine ear model

**Figure 3:**
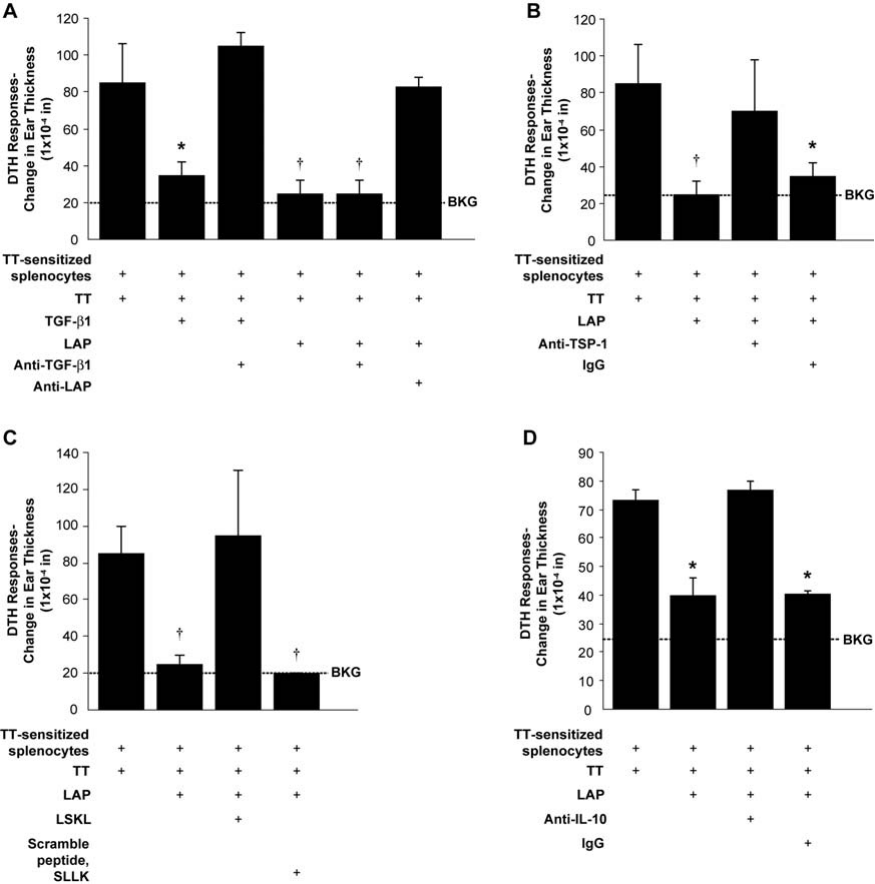
LAP is able to suppress *in vivo* cellular inflammation similar to TGF-β1 through interactions with thrombospondin-1. A transfer DTHR assay was performed with antigen (tetanus toxin) and tested agents (TGF-β1, rhLAP and anti-TGF-β1, LAP or TSP-1). C57BL/6 mice were sensitized to tetanus toxin (TT) and then tested to confirm they had developed a TT-induced DTHR. DTHR was induced in the pinnae of naïve C57BL/6 mice with simultaneous injection (35 µl) of antigen and splenocytes (8×10^6^ cells/condition) harvested from antigen-sensitized mice. DTHR was quantified by measurement of ear thickness 24 hours after injection. (A) LAP (10 pg) and TGF-β1 (5 ng) were tested for the ability to reduce DTHR when used alone, with anti-TGF-β1 antibodies (1 µg) or anti-LAP antibodies (1 µg) (*p<0.02 versus tetanus sensitized splenocytes + TT; †p<0.01 versus tetanus sensitized splenocytes + TT, n = 5). Similar results were observed when this assay was repeated using C57BL/6 mice that had rejected a cardiac allograft (DBA/2->C57BL/6) as a model of transfer DTHR. (B) Anti-TSP-1 antibody or isogenic control IgG (all at 1 µg) were tested for their ability to interfere with rhLAP suppression of TT-induced DTHR (*p<0.02 versus tetanus sensitized splenocytes + TT; †p<0.01 versus tetanus sensitized splenocytes + TT, n = 3). (C) Transfer DTHR assay was performed with antigen, rhLAP and the inhibitory peptide, LSKL or its scrambled control SLLK (20 µM) (†p<0.01 versus tetanus-sensitized splenocytes + TT, n = 2). (D) To determine if LAP used an IL-10 dependent pathway to inhibit the DTHR (as previously published) the DTHR transfer assay was repeated with anti-IL-10 antibodies and isotype control. (1 µg each) (*p<0.01 vs. tetanus-sensitized splenocytes + TT, n = 3) All bars represent the mean±SEM. DTHR, delayed-type hypersensitivity response; Ag, Antigen; BKG, background ear girth.

TGF-β1 has well characterized immunosuppressive effects on leukocytes.[7], [27], [28] To determine if LAP mediated immunosuppressive effects *in vivo*, experiments were designed to establish if LAP independently affected cellular inflammation. Using a well-characterized murine model of allogeneic delayed-type hypersensitivity (DTHR) inflammation of the pinnae, both recombinant human (rh) LAP and (rh) active TGF-β1 reduced inflammation measured by ear swelling (p<0.001 for either treatment versus vehicle control) (Figure 3A). While TGF-β1 inhibits DTHR in this murine model,[20] finding that LAP mimicked these effects is novel. Furthermore, anti-LAP IgG reversed LAP-mediated suppression of DTHR (p<0.01) whereas anti-TGF-β1 antibodies or control IgG did not (Figure 3A).

Based on the pathways found to be important in LAP-mediated monocyte recruitment, the role of TSP-1 in LAP-induced inhibition of DTHR was explored. Anti-TSP-1 antibodies were injected along with antigen into sensitized animals. TSP-1 antibodies reversed LAP-induced inhibition of DTHR (p<0.01) while control IgG did not (Figure 3B). Importantly, this effect was reliant on LAP, because when antigen and anti-TSP-1 antibodies were injected locally without LAP, no effect was seen in the elicited DTHR (data not shown). In separate studies, the peptide, LSKL reversed the effects of LAP on DTHR (p<0.01) (Figure 3C), while equivalent concentrations of the scrambled peptide SLLK did not. CD36 appeared to play less of a role in this immunologic effect as anti-CD36 antibodies only showed a trend toward interference with LAP-mediated inhibition of DTHR, and the reversal appeared incomplete [DTH thickness 61± 2×10^−4^in (control) vs. 19±3×10^−4^in (LAP alone) vs. 28±2×10^−4^in (LAP+ anti-CD36 Ab), p = 0.058].

Previous work has shown that IL-10 and TGF-β1 are critical mediators of the suppression of the DTH response in this experimental system.[29] While inhibiting TGF-β1 with a blocking antibody (Figure 3A) did not change the LAP effect, co-incubation with IL-10 blocking antibodies prevented LAP from blocking the DTH response (p<0.01, Figure 3D). In contrast, isogenic antibodies did not interfere with the effects of LAP on DTHR. This data suggests that IL-10 is a critical downstream effector of this observed LAP effect.

### Macrophages and mice lacking TSP-1 are insensitive to the immune regulatory effects of LAP

**Figure 4:**
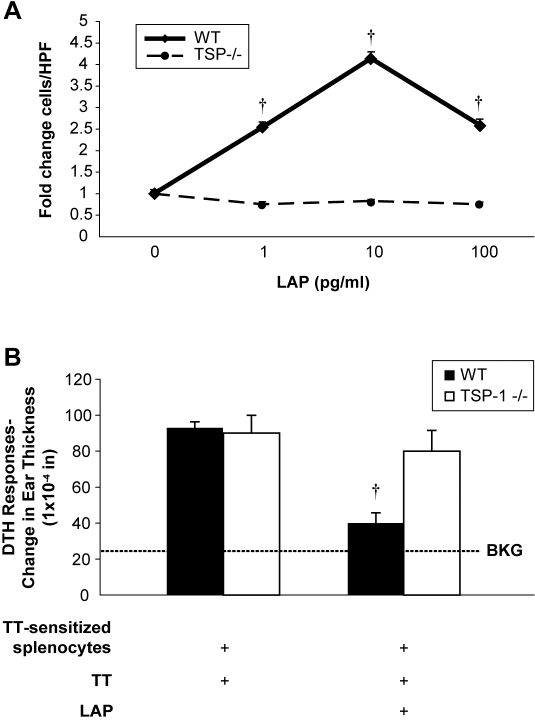
LAP-induced inflammatory cell recruitment is altered in TSP-1 deficient (TSP-1 −/−) mice. (A) Bone marrow macrophages from wild type C57BL/6 or TSP-1−/− mice were isolated and assessed for chemotaxis activity using modified Boyden chambers. Macrophages (5×10^4^/condition) were suspended serum-free in Gey's balanced salt solution and chemotaxis was assayed by counting the mean of five blinded high-powered fields on the polycarbonate filter (†p<0.001 for the chemotaxis of wild-type vs. TSP-1 −/− BMM). The data is expressed as fold-change in the mean compared to control (media alone)±SEM for two independent studies done in triplicate. (B) LAP's ability to affect the direct DTHR was tested in TSP-1 −/− and wild-type mice. TSP-1 −/− and wild-type C57BL/6 mice were sensitized to tetanus toxin (TT) and then tested to confirm they had developed a TT-induced DTHR. This direct DTHR assay was performed in the pinnae of these mice with simultaneous injection (35 µl) of antigen (tetanus toxin) alone or with rhLAP (10 pg) (†p = 0.004 for LAP-treated versus untreated WT mice, n = 3). All bars represent the mean±SEM for two studies unless otherwise noted. DTHR, delayed-type hypersensitivity response; TT, tetanus toxin; WT, wild type, BKG, background ear girth.

To confirm that TSP-1 was essential to the biological function of LAP, bone marrow-derived macrophages (BMM) from TSP-1 deficient or wild type mice were utilized in chemotaxis assays to assess their responsiveness to LAP. BMM from C57Bl/6 wild-type mice with functional TSP-1 migrated towards rhLAP (p<0.001) (Figure 4A). In contrast, BMM from C57Bl/6 *thrombospondin-1 −/−* mice had no significant increase in recruitment in response to LAP compared to control conditions (Figure 4A). Importantly, TSP-1 −/− macrophages responded to a known positive stimulus.

To determine the contribution of TSP-1 to the *in vivo* effects of LAP, TSP-1 deficient and wild type mice were evaluated for an LAP-mediated reduction in the allogeneic DTHR in the pinnae. LAP did not inhibit tetanus-induced DTHR in TSP-1 −/− mice (p = 0.678), but reduced ear swelling in wild-type control mice from the same background (p = 0.004) (Figure 4B), suggesting the importance of TSP-1 to the biological activity of LAP.

## Discussion

Data presented in this manuscript demonstrates that LAP has independent biological effects in modulating monocyte recruitment both *in vitro* and *in vivo*, and suppressing DTHR in a murine ear model. LAP promoted the chemotaxis of human monocytes through a TSP-1 and CD36-mediated pathway; whereas, LAP suppression of DTH was more dependent on TSP-1 and IL-10. These observations recapitulate some of the biological properties ascribed to the TGF-β1 molecule itself suggesting a potentially novel role for LAP in regulating cellular inflammation. In fact, as suggested by our chemotaxis data, TGF-β1 inhibited monocyte chemotaxis induced by LAP, suggesting a potential role for TGF-β1 in suppressing LAP-mediated cellular activation.

*In vitro,* LAP required TSP-1 and CD36 to recruit monocytes, while other ligands that interact with TSP-1, like RGD peptides and CD47, did not appear to mediate these responses. *In vivo* LAP utilized an IL-10-dependent pathway to mediate suppression of the cellular immune response. The importance of IL-10 in the cellular DTH response has been demonstrated in this model[29] and its connection to an LAP-initiated process provides an explanation of how LAP could both induce cellular recruitment and reduce inflammation. We are focusing on defining the cell of origin and the mechanism by which LAP involves IL-10 in this *in vivo* model. It is possible these effects could be mediated through local dendritic cells, as dendritic cell exposure to LAP has been shown to influence T-cell production of IL-10.[14]

The concept of an independent role for LAP in immune homeostasis has precedent. Investigators have proposed that the large latent complex of TGF-β1 (TGF-β1+LAP+LTBP1) is not just a passive sink for active TGFβ1, but rather an active sensor of the matricellular environment.[10] While cellular recruitment regulated by LAP may not be a direct part of this regulatory paradigm, our data supports this view. In fact, the early and late effects of TGF-β1 in some model systems could be interpreted as the sum of the actions of TGF-β1 *plus* the independent effects of LAP.

Several studies indicate that LAP may also be important in pathophysiologic processes. Experiments in a murine model of human scleroderma and GVHD indirectly support LAP as an independent regulator of immune cell function. In these studies, bone-marrow transplantation was performed in mice to induce TGF-β1-mediated skin fibrosis and inflammation.[5] The investigators asked if systemic therapy with recombinant LAP abrogated the development of skin fibrosis in this animal model. While the data showed an expected reduction in TGF-β1 signaling and fibrosis, there was persistent monocytic inflammation in the skin. Since monocyte recruitment can be a function of TGF-β1 or LAP, these data suggest that LAP could have had independent biological activity in this model. Other organ effects were not reported in these studies. The authors concluded that LAP inhibits the portion of known TGF-β1 signals that result in fibrosis, but that TGF-β1 likely signaled through other pathways to mediate inflammation. However, independent effects of LAP were not considered.

Recently, a genetic abnormality affecting the function of LAP was described as the underlying cause of a naturally occurring human disease called Camurati-Engelmann's disease (CED). CED is a disorder associated with abnormal bone growth and multiple other systemic abnormalities.[30] Two groups recently reported that this disorder was associated with several missense mutations in the LAP region of the TGF-β1 gene that affects its ability to bind to TGF-β1 and confer latency.[31] It is possible that some of the phenotypic abnormalities in CED patients may be related to changes in LAP and resultant downstream signaling events.

Our data indicate that the type I repeat region (TSR) of TSP-1 is important to the biological effects of LAP. The associations between the TSR regions of TSP-1 and LAP have been well studied and characterized.[32]–[34] These associations, acting specifically through the LSKL region of LAP are known to activate latent TGF-β1 in cell or cell-free systems.[25] Recent studies clarify how these two molecules interact to effect the dynamic conformational changes that occur during activation of latent TGF-β1. L^54^SKL in LAP binds to an R^94^KPK region of the active TGF-β1 molecule in the small latent complex.[34] In a proposed model of TSP-1-mediated activation of latent TGF-β1, the TSR binds to a VLAL region on the active TGF-β1 molecule allowing the K^412^RFK motif on TSP-1 to compete for binding with L^54^SKL.[33] Adding complexity to the role of TSP-1 in TGF-β1 activation is the finding that K^412^RFK residues on TSP-1 are also critical for the biological function of LAP, which is disrupted by L^54^SKL[25]

Our inhibitor data support the secondary hypothesis that TSP-1 and CD36 are important for some aspects of LAP-mediated immune cell regulation. This is not surprising as TSP-1, TGF-β1 and CD36 are intimately connected physiologically.[32] Finding that peptides against the type I repeat region of TSP-1 inhibited LAP-induced cellular activation confirmed that TSP-1 is critical to the biological functions of LAP. Blocking CD36, a known receptor for TSP-1, limited the LAP-mediated effects in a chemotaxis model of monocyte recruitment. However, blocking CD36 did not appear as integral to LAP's ability to block allogeneic DTHR as there appeared to only be a trend toward modest attenuation in this system. Our data suggest that the signaling induced by LAP is more complex than a simple interaction between LAP and CD36 mediated by the type I repeat region of TSP-1. This is based on the fact that the anti-TSP-1 antibody used to inhibit LAP function in these studies was the clone A6.1, which targets the C-terminal collagen type V binding region of TSP-1. Although the exact binding region of TSP-1 that is targeted by clone A6.1 is not known, because of the close proximity of the type I repeat region to the C-terminal domain of TSP-1 and the size of the antibody it is plausible to think the antibody's presence may interfere with type I repeat function. Since RGD peptides or anti-CD47 IgG did not modify the biological activity of LAP, we presume that the inhibition of LAP function by clone A6.1 was not due to blocking CD47 or RGD peptide binding regions. Regardless, it is likely that more than the type I repeat region of TSP-1 is necessary for LAP to induce target cell activation. TSP-1 may anchor several proteins together to facilitate signaling as has been shown in other experimental systems.[35], [36]

In summary, this report demonstrates that LAP has biological activity independent of its parent molecule, TGF-β1, that requires TSP-1 and to a lesser extent CD36 in human monocytes. These data provide important support for a critical re-evaluation of the relationship of LAP to active TGF-β1and its role in biologic systems. This hypothesis emphasizes the need to understand the “fate” of the LAP after pathophysiologic activation of TGF-β1 and may support investigating it as a novel biological target for fibrotic and inflammatory human disease.
